# High potency fish oil supplement improves omega-3 fatty acid status in healthy adults: an open-label study using a web-based, virtual platform

**DOI:** 10.1186/1475-2891-12-112

**Published:** 2013-08-08

**Authors:** Jay K Udani, Barry W Ritz

**Affiliations:** 1 Medicus Research, 28720 Roadside Drive, Suite 310, Agoura, CA 91301, USA; 2 2Atrium Innovations Inc, 4 Hillman Drive, Suite 190, Chadds Ford, PA 19348, USA; 3 3Nutrition Sciences Department, College of Nursing and Health Professions, Drexel University, 245 N. 15th Street, Philadelphia, PA 19102, USA

**Keywords:** Omega-3 index, Eicosapentaenoic acid, Docosahexaenoic acid, Fish oil, Open-label

## Abstract

**Background:**

The health benefits of omega-3 fatty acids from fish are well known, and fish oil supplements are used widely in a preventive manner to compensate the low intake in the general population. The aim of this open-label study was to determine if consumption of a high potency fish oil supplement could improve blood levels of eicosapentaenoic acid (EPA) and docosahexaenoic acid (DHA) and impact SF-12 mental and physical health scores in healthy adults.

**Methods:**

A novel virtual clinical research organization was used along with the HS-Omega-3 Index, a measure of EPA and DHA in red blood cell membranes expressed as a percentage of total fatty acids that has been shown to correlate with a reduction in cardiovascular and other risk factors. Briefly, adult subjects (mean age 44 years) were recruited from among U.S. health food store employees and supplemented with 1.1 g/d of omega-3 from fish oil (756 mg EPA, 228 mg DHA, Minami Nutrition® MorEPA® Platinum) for 120 days (n = 157).

**Results:**

Omega-3 status and mental health scores increased with supplementation (p < 0.001), while physical health scores remained unchanged.

**Conclusions:**

The use of a virtual, web-based platform shows considerable potential for engaging in clinical research with normal, healthy subjects. A high potency fish oil supplement may further improve omega-3 status in a healthy population regularly consuming an omega-3 supplement.

## Introduction

Considerable evidence has demonstrated that increasing consumption of omega-3 fatty acids can benefit health, most notably by reducing cardiovascular disease and its associated risk factors [1-5]. Over the past several decades, evidence has emerged indicating that higher intakes of omega-3 are associated with a significant reduction in all-cause mortality [6,7], and a 2006 meta-analysis of randomized controlled trials found that fish oil significantly reduced total mortality [8]. Specifically, eicosapentaenoic acid (EPA) and docosahexaenoic acid (DHA) have been identified as health promoting. Both EPA and DHA may improve cardiovascular health by several mechanisms. In general, both EPA and DHA compete with arachidonic acid (omega-6) in cyclooxygenase and lipoxygenase synthesis, resulting in net anti-inflammatory effects. Both reduce blood viscosity without a significant effect on platelet or clotting factors, and both have positive effects on blood lipids, most consistently a reduction in triglyceride concentrations [9]. However, some recent analyses have suggested that protection against cardiovascular outcomes may not be as pronounced as indicated by earlier investigations [10,11], raising awareness that additional controlled studies are needed.

Data are also emerging connecting omega-3 fish oils with cognitive function and mental health status [12-16]. Population studies and preliminary clinical data demonstrate that omega-3 fatty acids may help elevate mood in depressed subjects [13,14]. In particular, high-EPA interventions and higher plasma EPA may be associated with improved clinical outcomes [17], although more research is needed in the application of EPA and fish oil in cognitive and mental health.

Despite the clear need for more controlled studies in specific populations and targeting specific clinical outcomes, increasing blood EPA and DHA levels appears to have a generally positive impact on health, and awareness among the general population is high [18]. Because of this, the use of fish oil supplements has increased dramatically over the past decade, reaching sales of $1.145 billion in 2011 and continuing to grow [19]. The American Heart Association recommends an intake of 1 g/d of combined EPA and DHA from food or supplements for secondary prevention [20], and concentrated formulations of EPA and DHA are now available as both dietary supplements and medications.

The aims of the present study were to investigate the utilization of a new web-based program known as a virtual clinical research organization (CRO) and to assess the effects of a high potency fish oil on omega-3 status, as well as mental and physical health scores, in healthy adults who regularly consume an omega-3 supplement. To our knowledge, this represents the first such use of a virtual CRO to assess the impact of a nutritional intervention on a quantifiable biomarker of health in the general population.

## Materials and methods

### Virtual contract research organization (CRO)

The proprietary, web-based CRO program was developed by Medicus Research (Northridge, CA). This system enabled researchers to enroll subjects through inclusionary and exclusionary dialogue and track and monitor progress by using survey tools and automatic prompts. Researchers virtually followed up with subjects regarding completion of milestone events, adverse event reporting, non-compliance issues, and other study requirements.

### Subject recruitment and protocol

The protocol was approved by Western IRB (Olympia, WA) prior to the initiation of any study related activities. Healthy subjects between the ages of 18 and 70 years working at health food stores nationwide were recruited. Subjects (n = 316) were invited via email to participate in the study. Those who accepted the offer were directed to a secure website where they took part in an online informed consent procedure with electronic signature. Participants then completed questions related to standard inclusion and exclusion criteria such as age (18–70 years), agreement to participate, and exclusion based on a history of bleeding disorders, current use of anti-coagulant medications, immune disorders, diabetes, planned surgery, or pregnancy, among other criteria. The subjects agreed to not change their current diet or exercise program and to stop using all other dietary supplements, including their present omega 3 fish oil supplement, for a two-week wash-out period before onset of the study and for the duration of the intervention. Subjects were then scheduled to undergo a phone screen with a trained clinical staff member. This phone screening also ensured the subjects understood their rights and responsibilities related to the study prior to their participation.

The study duration was 120 days and featured a total of 5 virtual CRO visits (V1-5). The subjects answered a series of online questionnaires regarding medical history, including an assessment of current supplemental omega-3 intake and web-based SF-12 Health Survey (V1). Subjects then received via mail the first month’s supply of the study product and the Omega-3 Index test kit, which was to be completed on the same day they began taking the study product. The study product provided a total of 1,100 mg omega-3 s with 756 mg EPA, 228 mg DHA, and 300 IU vitamin D3 in a single soft gel capsule (Minami Nutrition® MorEPA® Platinum, provided by Garden of Life, West Palm Beach, FL).

The subjects continued to receive monthly study product via mail, as well as an email reminder from the Virtual CRO for the 30-day (V2), 60-day (V3), 90-day (V4), and 120-day (V5) assessments. The emails directed the subjects to a secure link for completion of the compliance assessment, medical review, SF-12 Health Survey, and adverse event questionnaires. The subjects were also instructed to complete the Omega-3 Index test at V5. Compliance was assessed through the study website where subjects were instructed to enter the number of pills remaining in the study product bottle at the end of each month.

### Omega-3 status

Omega-3 status was quantified using the HS-Omega-3 Index, a finger stick blood test that measures the amount of EPA and DHA in red blood cell membranes expressed as the percentage of total fatty acids in the membrane. The test was employed according to manufacturer’s instructions and results were analyzed and reported by the manufacturer (OmegaQuant, Sioux Falls, SD). Briefly, subjects received test kit directly from the laboratory responsible for testing, which includes a large bag, small bag, test request form, collection card, alcohol pad, adhesive bandage, cotton ball, lancet, desiccant pack, and return envelope. Subjects were instructed to fill out test request form and collection card first and wash hands thoroughly before sterilizing either ring or middle finger with the alcohol pad. Subjects used lancet on sterilized finger and applied drop of blood directly onto collection card, blood was allowed to dry on the card for 15–20 minutes, and card was then folded in half over the spot and placed in the small bag with desiccant. The small bag was placed in the large bag along with the test request form, and then the large bag was placed in return envelope. Subjects were instructed to return the envelope via mail on the same day as collection. The data management team received the results.

### SF-12 health survey

The subjective assessment of mental and physical health as composite scores utilized a shortened, validated version of the SF-36 health survey created by Quality Metric and in use since 1994 [21-23]. The scoring system applied was from Positive Aging Resource Center, and the reference range of scores had a cutoff of 50 for Physical Component Summary (PCS) and 42 for Mental Component Summary (MCS).

### Statistical analysis

All values are reported as the mean ± the standard error of the mean (SEM) or as a percentage of total subjects, and data were analyzed using SPSS (Chicago, IL). Paired T-tests were used to test significance for the HS-Omega 3 Index (baseline versus day 120), and analysis of variance (1 by 4) was used to determine time effect of the intervention on the SF-12 composite scores.

## Results

A total of 316 subjects were recruited for the study, 251 subjects completed a baseline HS-Omega-3 Index assessment, and 157 subjects completed all aspects of the trial through 120 days. Non-completers were lost to follow-up. Adverse events reported were not serious and were determined to not be associated with the study product. The mean age of subjects was 44.3 ± 0.9 years. Table 1 provides information on omega-3 supplement usage at baseline. No other demographic data were obtained.

**Table 1:** Baseline consumption rates of omega-3 fatty acid supplements

		**Fish oil capsules**	**Fish oil liquid**	**Vegetarian omega-3 capsules**
**Total users**^**1**^		66%	21%	19%
**Frequency of use**^**2**^	*1/d*	51%	72%	36%
	*2/d*	30%	3%	16%
	*3/d*	5%	1%	-
	*4/d*	2%	1%	-
	*5+/d*	1%	1%	-
	*1/wk*	11%	21%	47%
**Dosage**^**2**^	*< 500 mg*	10%	25%	63%
	*500–1000 mg*	57%	42%	26%
	*>1000 mg*	33%	33%	11%

^1^Percentage (%) of total study population.

^2^Percentage (%) of omega-3 supplement users by column.

**Figure 1:**
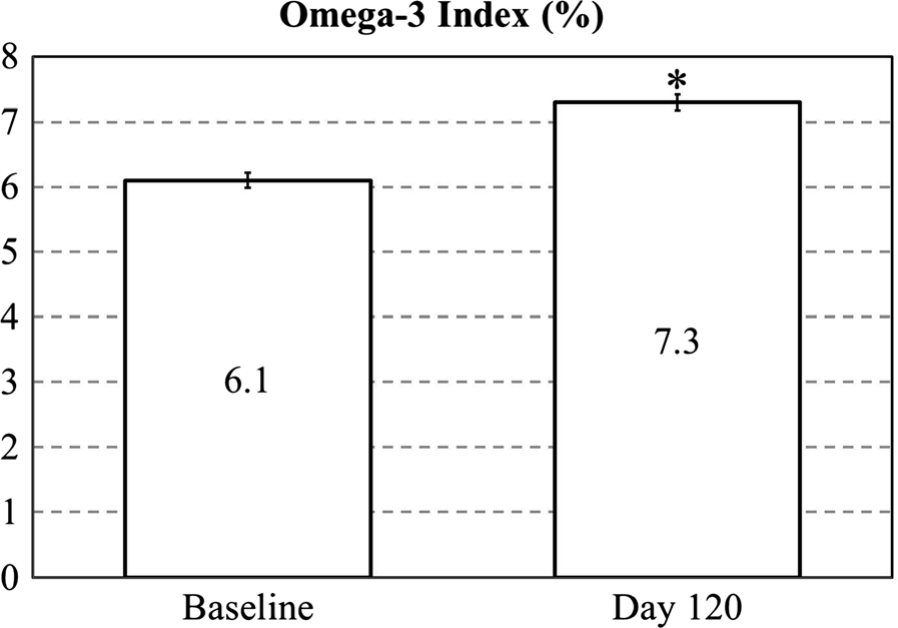
Omega-3 Index in healthy subjects at baseline and following fish oil supplementation, * *p < 0.001 *.

The HS-Omega-3 Index increased from 6.1% (range of 3.1-11.8%) at baseline to 7.3% (3.7-13.3%) at 120 days (p < 0.001, Figure 1). This represents an approximate 20% increase over baseline. The baseline score of approximately 6% was higher than previously reported baseline scores of approximately 4%, as expected, reflecting regular use of omega-3 fatty acid supplements [24,25]. The mental composite score increased after 60 days and remained elevated through day 120 (p < 0.001, Table 2). There was no change in the physical composite score at any time point during the intervention.

**Table 2:** Mental and physical health composite scores in healthy subjects at baseline and following fish oil supplementation

		**Days**		
	**Baseline**	**60**	**90**	**120**
**Mental health**	49.5 ± 0.5	52.5 ± 0.5*	53.0 ± 0.6*	53.5 ± 0.5*
**Physical health**	53.2 ± 0.3	53.1 ± 0.4	53.2 ± 0.4	53.86 ± 0.3

*p < 0.001 compared to baseline.

## Discussion

To our knowledge, the present study is the first to report a significant increase in blood EPA and DHA levels, as Omega-3 Index, in a group of healthy individuals who were regular consumers of fish oil supplements at baseline. Maximizing the response to omega-3 supplementation may be a prudent clinical target for the reduction of certain risk factors, and Omega-3 Index may be an appropriate biomarker for assessing and monitoring the effectiveness of omega-3 supplementation.

An increase in the Omega-3 Index has been correlated with reductions in cardiovascular risk factors, and low Omeg-3 Index has been reported as a predictor of sudden cardiac death [24-27]. Importantly, it has also been suggested that an Omega-3 Index of greater than 8% was associated with a lower risk for acute coronary syndromes and decreased morbidity and mortality in those with cardiovascular disease [28]. Conversely, an Omega-3 Index less than 4% was associated with a ten-fold increase in risk of cardiac death compared to subjects with an Omega-3 Index greater than 8% [29]. Inflammatory markers were also shown to be inversely related to Omega-3 Index [30], and an Omega-3 Index of 8% or higher was associated with slowed cellular aging as measured by the five-year rate of telomere shortening [31].

In the current study, we demonstrated a significant increase in Omega-3 Index in 120 days of regular supplementation with a high potency fish oil. Assuming a clinical target of >8%, the percentage of subjects meeting this target increased from 5.6% at baseline to 24.8% of subjects following supplementation. Such an increase may be expected to yield clinically relevant outcomes among responders, although this would require further study. It is unknown whether continued supplementation would further raise Omega-3 Index scores beyond what was observed at 120 days or increase the number of subjects achieving a score of >8%, although this seems a reasonable extrapolation of the current data.

The SF-12 health survey provides a quick assessment of an individual’s physical and mental health. The average baseline mental health and physical health scores from this study are similar to those previously reported [32]. In this study, we also detected a small but significant increase in mental health scores based on the SF-12 mental health composite compared to baseline. However, the study design did not include a placebo control, and there are insufficient corroborative data to conclude that omega-3 supplementation and a corresponding increase in Omega-3 Index can improve cognitive function in a healthy population.

The current study had certain limitations in design and execution. First, information was not obtained about the composition of omega-3 fatty acid products being consumed prior to the study, and only a basic analysis of omega-3 intake from food sources was performed (not reported). Subjects were instructed to maintain normal dietary habits, and fish consumption was not restricted. Further, it is possible that the subjects could have self-reported inaccurate information regarding their supplement use; however, over-reporting of supplement use is not likely as their omega-3 status was higher than what has been reported in the typical population. Next, the use of an open-label design creates the potential for bias by both investigator and subject. The virtual CRO is designed to track and monitor the subject compliance with minimal human interaction and influence. While this is a positive, the tradeoff may be in overall compliance. While the absence of a placebo does not allow for the determination of normal changes in blood EPA and DHA levels associated with daily nutritional practices, because the subjects had already been taking a fish oil supplement, the baseline value served as the surrogate placebo or comparator value. Further, it was impractical and perhaps unethical, given the known health benefits of omega-3 consumption, to restrict omega-3 supplementation in a currently supplementing healthy population for a prolonged period of 4 months. Therefore, it was determined that a placebo was not required. Clearly, the lack of a placebo arm does not allow for the assessment of causality in the subjective assessment of mental health scores.

## Conclusion

Overall, the combination of the virtual CRO method, the use of the SF-12, and the self-administered Omega-3 Index finger prick blood test provided an appropriate, effective, and efficient design for assessing the potential impact of a nutritional supplement on biomarkers of health in a non-diseased population. The current study demonstrated significant improvements in blood EPA and DHA status, and a small but significant increase in subjective mental health scores, following the compliant use of a high potency fish oil supplement in a healthy population that had been previously taking a fish oil supplement. Further studies are needed with more rigorous design, extended duration, and the evaluation of clinical endpoints to assess the relevance of high potency fish oil supplementation in the general population. Additional studies should evaluate the effectiveness of the virtual CRO as a research tool in evaluating nutritional interventions in a healthy population, as such studies are grossly lacking.

## Competing interests

The study was sponsored by Garden of Life, West Palm Beach, FL, a subsidiary of Atrium Innovations, Inc. Medicus Research conducted the study independent from the sponsor, such that the sponsor had no influence on the outcome of the study.

## Authors’ contributions

Both authors conceived of the design. JKU oversaw the execution of the study and data analysis including statistical analysis. Both authors read and approved the final manuscript.
